# Epigenetic Reprogramming of Erythroid Progenitor Cells: Insights for Enhancing Cancer Immunotherapy

**DOI:** 10.7150/ijbs.127543

**Published:** 2026-01-22

**Authors:** Zi-Zhan Li, Xuan-Yu Su, Cheng-Ke Zhou, Su-Ran Li, Zhi-Jun Sun

**Affiliations:** The State Key Laboratory of Oral & Maxillofacial Reconstruction and Regeneration, Key Laboratory of Oral Biomedicine Ministry of Education, Hubei Key Laboratory of Stomatology, School & Hospital of Stomatology, Frontier Science Center for Immunology and Metabolism, Taikang Center for Life and Medical Sciences, Wuhan University, Wuhan, 430079, China.

**Keywords:** cancer, erythroid progenitor cells, immunotherapy, epigenetic modifications

## Abstract

Cancer immunotherapy has markedly improved clinical outcomes for cancer patients. However, its broad application is constrained by low response rates, which limit therapeutic benefits to only a subset of individuals. A deeper understanding of the tumor microenvironment (TME) and the interactions between tumor and immune cells is crucial for overcoming resistance. In this context, the reprogramming of erythroid progenitor cells (EPCs) within the TME has emerged as an important mechanism of immunotherapy resistance. EPCs, a key population in erythroid differentiation, undergo epigenetic reprogramming that underlies various physiological and pathological states. Through epigenetic modifications, EPCs may interact with immune cells and thereby promote tumor immune evasion. This review summarizes EPC reprogramming in the TME from an epigenetic perspective and explores their crosstalk with tumor and immune cells. It also evaluates the therapeutic potential of epigenetic drugs targeting EPCs and discusses future research directions focused on reversing pathological epigenetic reprogramming in EPCs to enhance immunotherapy efficacy. These advances hold significant potential for optimizing clinical cancer care paradigms and improving patient prognosis.

## 1. Introduction

Cancer has emerged as the most severe public health challenge worldwide, accounting for one-sixth of all global deaths and imposing a disproportionately heavy burden^1^, ^2^. Cancer clinical management encompasses both medical interventions and supportive care, employing multidimensional therapeutic strategies. Particularly noteworthy is immunotherapy, an emerging therapeutic approach that has revolutionized cancer treatment paradigms^3^. However, response rates remain limited to 10-25% of patients, with many initially responsive cases eventually developing acquired resistance^4^-^6^. Enhancing the efficacy of immunotherapy holds significant promise for extending cancer patient survival. Tumor microenvironment (TME) plays a crucial role in determining immunotherapy outcomes through its complex interactions between tumor cells, stromal components, vasculature, and immune cells^7^-^9^.

Recent research has identified erythroid progenitor cells (EPCs) as particularly potent immunosuppressive components within the TME, potentially exceeding the immunosuppressive effects of myeloid-derived suppressor cells (MDSCs), tumor-associated macrophages (TAMs), and regulatory T cells (Tregs) in certain human tumor models^10^, ^11^. These progenitor cells, which exhibit abnormal expansion and differentiation arrest under tumor conditions, mediate immunosuppression through multiple pathways including reactive oxygen species (ROS) production, arginase-1 (ARG-1)-mediated arginine depletion, and secretion of inhibitory cytokines like transforming growth factor-beta (TGF-β)^10^, ^12^-^14^. Their prominent role in tumor immunosuppression positions EPCs as promising therapeutic targets to overcome immunotherapy resistance.

Epigenetics focuses on heritable changes in gene expression that occur without altering the DNA sequence, primarily mediated through DNA methylation, histone modifications, and non-coding RNA regulation^15^. These mechanisms collectively establish the epigenetic landscape that determines cellular identity and function, playing central roles in cell differentiation and functional plasticity^16^. The lineage commitment of EPCs, determining whether they differentiate into normal oxygen-transporting erythrocytes or adopt immunomodulatory functions within the TME, is precisely regulated through sophisticated epigenetic mechanisms. Recent years have witnessed remarkable progress in epigenetic research in oncology, successfully elucidating the crucial roles of various epigenetic modifications in tumor initiation and progression^17^, ^18^. Large-scale multi-omics studies have established the central importance of aberrant DNA methylation, imbalanced histone modifications, and chromatin remodeling defects in driving tumor cell proliferation, immune evasion, and drug resistance. Furthermore, dysregulated expression of non-coding RNAs (ncRNAs), particularly microRNAs (miRNAs) and long non-coding RNAs (lncRNAs), has been shown to indirectly alter transcriptional profiles by modulating epigenetic enzyme expression, thereby creating a pro-tumorigenic microenvironment^19^.

This review examines the differentiation pathways of EPCs in both physiological and pathological contexts, with particular emphasis on their functional characteristics within the TME. This review focus on the regulatory roles of epigenetic mechanisms including DNA methylation, histone modifications, and ncRNAs in determining EPCs fate and their contribution to immunosuppression and tumor progression. By synthesizing current advances in cancer epigenetics, we analyze the therapeutic potential of targeting epigenetic dysregulation in EPCs and discuss potential drug candidates and intervention strategies. This review is to establish a conceptual framework for understanding the epigenetic networks governing EPCs in cancer and to explore epigenetically targeted approaches that may ultimately enhance clinical outcomes for cancer patients.

## 2. EPC: An Emerging Immunosuppressive Population in the TME

**Figure 1:**
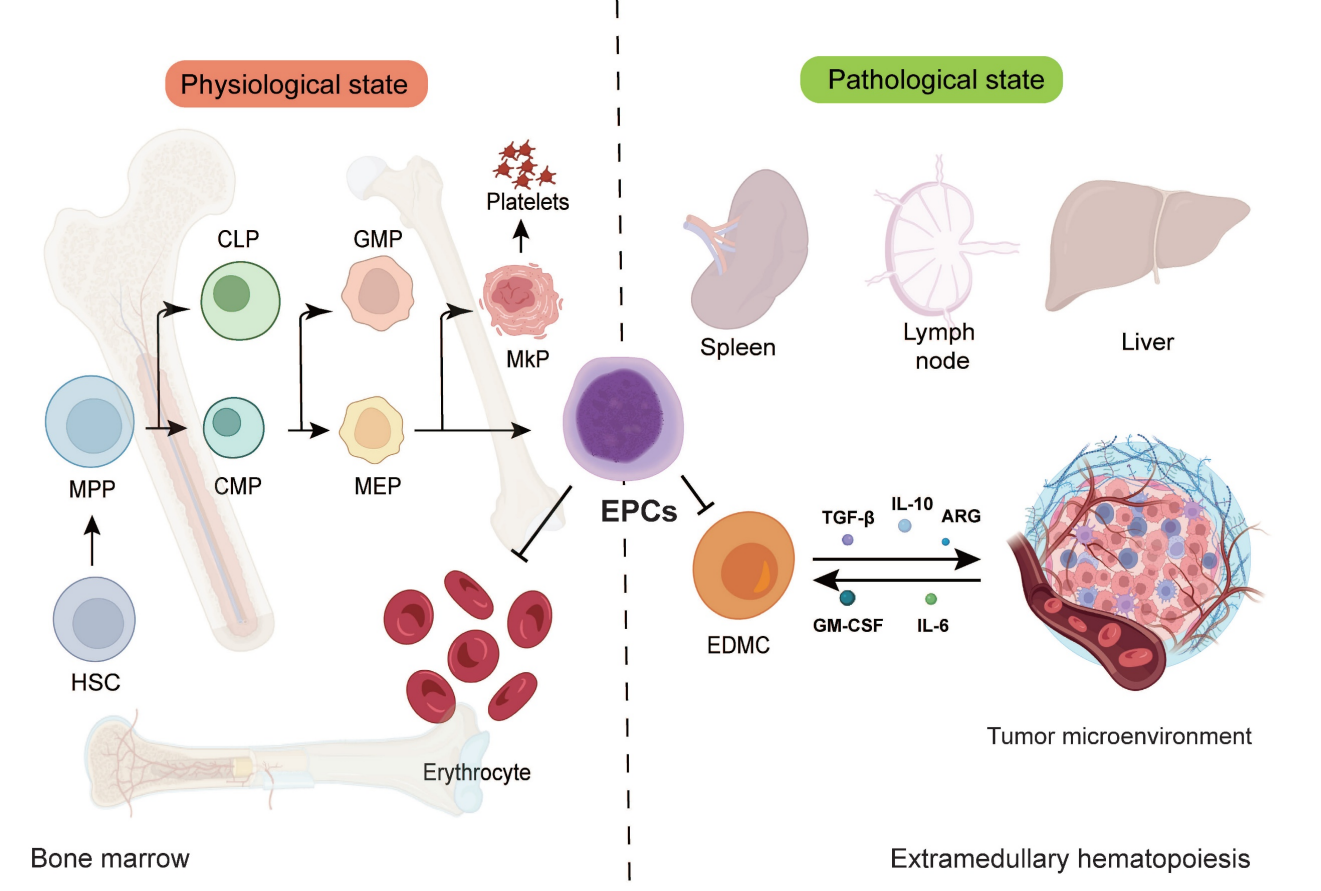
** EPCs maturation and differentiation​**​. EPCs represent a functionally distinct subpopulation within the erythroid differentiation hierarchy. These cells emerge primarily in the splenic environment and engage in EMH under pathological conditions. Notably, EPCs possess the capacity to migrate into the TME, where they actively participate in tumor development and progression. EPC, erythroid progenitor cell; HSC, hematopoietic stem cells; MPP, multipotent progenitor; CLP, common lymphoid progenitor; CMP, common myeloid progenitor; GMP, granulocyte-macrophage progenitor; MEP, megakaryocyte-erythroid progenitor; EDMC, erythroid-derived myeloid cell.

EPCs represent a crucial cellular population in erythropoiesis that has garnered increasing attention in tumor immunology in recent years^20^. These cells possess the potential to differentiate into erythrocytes and play pivotal roles in hematopoiesis, with their functional status directly influencing both the quantity and quality of erythrocyte production, thereby affecting systemic oxygen transport and physiological functions^21^. Beyond their normal erythroid differentiation, EPCs actively participate in shaping the TME and modulating immune responses under pathological conditions. EPCs originate from hematopoietic stem cells (HSCs), which possess self-renewal capacity and multilineage differentiation potential, serving as the progenitor of all blood cell types^22^. During early hematopoiesis, HSCs first give rise to multipotent progenitors (MPPs), which subsequently differentiate into either common lymphoid progenitors (CLPs) or common myeloid progenitors (CMPs)^23^. CLPs belong to the lymphoid lineage, generating T cells, B cells, and natural killer (NK) cells, while CMPs belong to the myeloid-erythroid lineage, producing granulocyte-macrophage progenitor (GMPs) and megakaryocyte-erythroid progenitors (MEPs)^24^. MEPs serve as the direct precursors of EPCs, capable of differentiating into either megakaryocytes (producing platelets) or erythroid lineage cells. **Figure 1** illustrates the maturation and differentiation processes of EPCs.

Under physiological conditions, EPCs primarily develop in the bone marrow. However, pathological states such as cancer, chronic inflammation, and hypoxia can induce extramedullary hematopoiesis (EMH), generating EPCs in organs including the spleen, liver, and lymph nodes. EMH represents a compensatory mechanism when bone marrow function is compromised or erythrocyte demand increases^25^, ^26^. In tumor-associated EMH, the spleen frequently exhibits abnormal EPCs expansion correlated with immunosuppressive phenotypes. Furthermore, studies demonstrate that perivascular pericytes in solid tumors can locally generate erythroid progenitors when expressing platelet-derived growth factor-B (PDGF-B), suggesting tumors can directly regulate EPC production and function through local hematopoietic niches^27^. Following differentiation from MEPs, EPCs progress through sequential developmental stages: early-stage burst-forming unit-erythroid (BFU-E) cells remain mostly quiescent as an erythroid reserve, while later colony-forming unit-erythroid (CFU-E) cells proliferate rapidly and mature through proerythroblast, basophilic erythroblast, polychromatophilic erythroblast, and orthochromatic erythroblast stages before enucleating into mature erythrocytes^28^.

Within the TME, EPC differentiation frequently becomes disrupted. Various cytokines and chemokines such as GM-CSF and IL-6 secreted by tumor cells can alter EPC differentiation trajectories, causing some EPCs to aberrantly transdifferentiate toward myeloid lineages, forming erythroid-derived myeloid cells (EDMCs)^29^. These EDMCs exhibit potent immunosuppressive capabilities through multiple mechanisms: expressing programmed death-ligand 1 (PD-L1), secreting immunosuppressive cytokines TGF-β and IL-10, and promoting Treg expansion. Moreover, EDMC accumulation has been shown to diminish the therapeutic efficacy of programmed cell death protein 1 (PD-1)/PD-L1 checkpoint inhibitors, representing a key mechanism underlying tumor immune therapy resistance.

## 3. Epigenetic Regulation of EPCs

**Figure 2:**
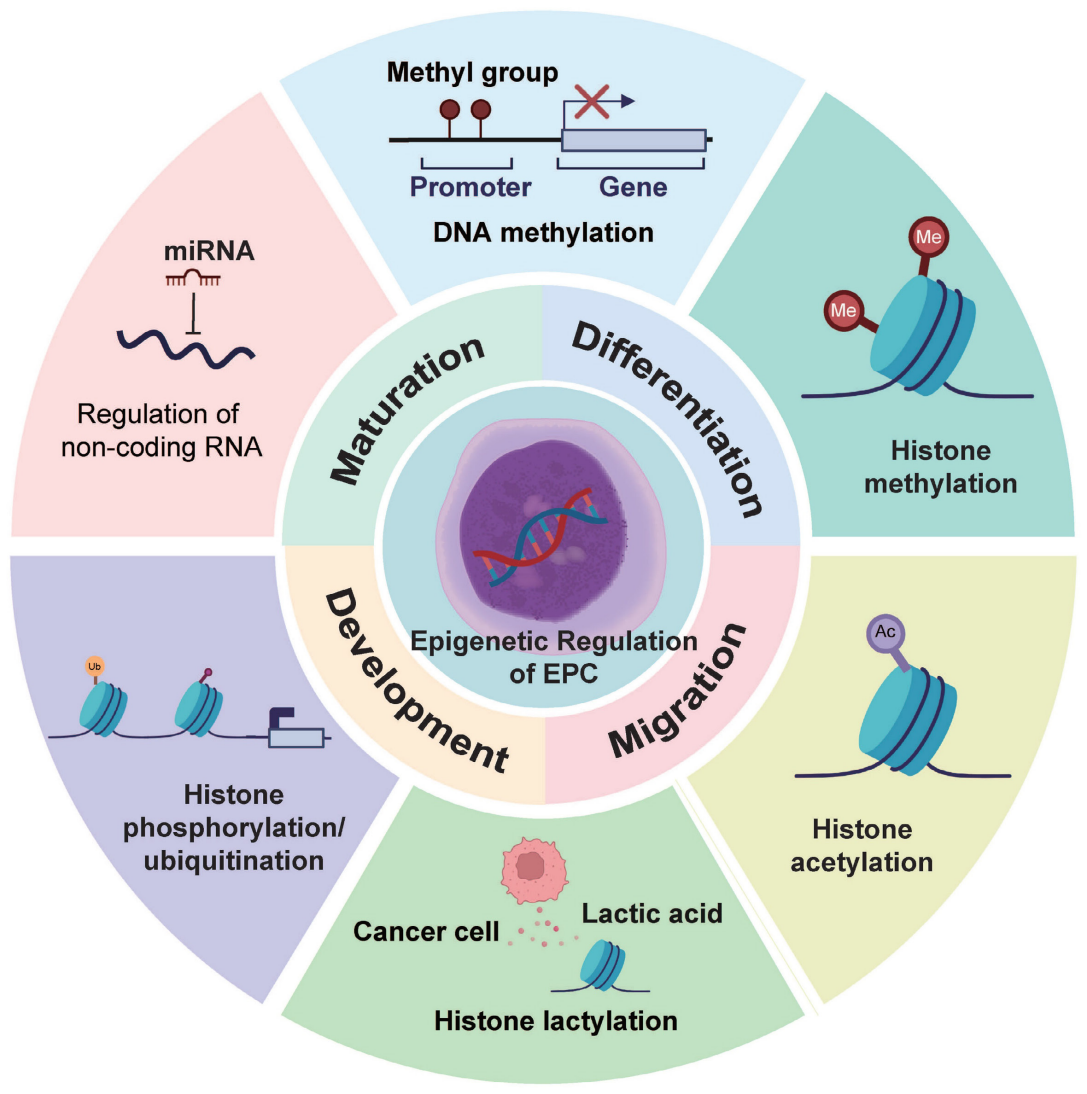
** ​​Epigenetic Reprogramming of EPCs​​.** EPCs exhibit diverse modes of epigenetic alterations. These epigenetic modifications influence the phenotypic characteristics of EPCs and may potentially shape the formation and properties of the TME.

EPCs represent a defined population in the erythroid differentiation hierarchy. However, their epigenetic dysregulation has been increasingly linked to the progression of various diseases and is widely investigated as a potential biomarker for disease advancement^30^. Although studies examining the epigenetic reprogramming of EPCs from an oncological perspective remain limited, the consequences of such reprogramming align closely with phenomena observed in the TME^31^. These epigenetic alterations may therefore modulate EPC-tumor cell interactions, thereby influencing cancer progression and tumor immune evasion. We summarize the major types of epigenetic modifications and their functions in EPCs in **Table 1** and** Figure 2**.

### 3.1 DNA Methylation​​

DNA methylation represents a core epigenetic mechanism regulating cell lineage differentiation, involving the addition of methyl groups to the 5-carbon position of cytosine residues (primarily within CpG dinucleotides) to modulate transcriptional states^32^, ^33^. During normal erythroid differentiation, DNA methylation patterns undergo dynamic remodeling: from HSPCs to mature EPCs, programmed genome-wide demethylation and remethylation at specific loci activate globin gene clusters while silencing non-erythroid genes (myeloid/lymphoid lineage transcription factors). This process is antagonistically regulated by DNA methyltransferases (DNMT1, DNMT3A, DNMT3B) and TET family dioxygenases (TET1/2/3), where DNMTs establish/maintain methylation marks, and TET proteins facilitate demethylation through hydroxymethylation and subsequent oxidation^34^. Loss of activation-induced cytidine deaminase (AID) induces myeloid cell expansion coupled with EPCs depletion, consequently leading to anemia and dysregulation of lineage-specific transcription factors *Cebpa* (myeloid) and *Gata1* (erythroid). Consistent with murine model data, AID silencing in human hematopoietic cells results in skewed differentiation toward myelomonocytic lineages and impaired erythroid maturation. AID deficiency alters transcriptional programs governing erythropoiesis regulators and promotes locus-specific DNA methylation, thereby modulating transcriptional regulation^35^. Moreover, the RNA N6-methyladenosine (m6A) methyltransferase METTL16 safeguards genomic integrity by orchestrating DNA repair-related genes, thereby coordinating DNA repair mechanisms in EPCs. METTL16-deficient erythroblasts exhibit impaired differentiation capacity alongside activation of DNA damage and apoptotic programs^36^.

The expression of the master erythroid regulator GATA1 is controlled by a specific methylation-determining region (G1MDR) within its promoter. Under hypermethylated conditions, DNMT1 maintains G1MDR silencing to suppress GATA1 transcription. Demethylation activates GATA1, initiating the erythroid gene expression network and driving terminal EPCs differentiation^37^. Within the TME, chronic inflammation, hypoxia, and tumor-secreted factors disrupt the DNMT-TET balance, causing aberrant DNA methylation programming. For instance, erythroid differentiation genes may remain abnormally hypermethylated and silenced, while promoters of ARG-1 and PD-L1 undergo hypomethylation and overexpression^38^, ^39^. These alterations trap EPCs in immature states with immunosuppressive phenotypes, enhancing TME-mediated immune evasion. Current studies further reveal similar hypomethylation-mediated activation patterns in other immunoregulatory genes (IDO1 and IL10) within TME-resident EPCs, correlating with MDSC-like functionality^32^, ^40^. Epigenetic agents targeting 5-azacytidine show preclinical potential to restore erythroid differentiation and attenuate EPCs immunosuppression.

### 3.2 Histone Modifications​​

Histone modifications constitute a class of covalent chemical alterations occurring at the N-terminal tails and core domains of histones, including acetylation, methylation, phosphorylation, ubiquitination, and SUMOylation^41^, ^42^. These modifications dynamically regulate chromatin conformation and transcriptional activity by altering histone-DNA interaction strength or providing binding sites for chromatin-associated factors. Histone acetylation typically associates with open chromatin configurations and transcriptional activation, while methylation at specific residues may either promote transcription (such as H3K4me3) or mediate repression (such as H3K27me3)^43^, ^44^. Interplay between distinct modifications forms intricate transcriptional regulatory networks through the histone code, playing pivotal roles in cellular differentiation, development, and disease pathogenesis. During EPC differentiation, histone modifications govern the expression patterns of key erythroid transcription factors including GATA1 and KLF1, as well as hemoglobin-related genes, thereby directing EPCs maturation and function. Cancer-associated inflammation and the tumor microenvironment disrupt normal EPCs differentiation trajectories through aberrant activation or suppression of specific histone modification pathways, exemplified by imbalances in histone acetyltransferase and histone deacetylase activities. Such dysregulation confers immunosuppressive phenotypes upon EPCs, facilitating their involvement in tumor immune evasion^45^. Consequently, histone modifications represent not only fundamental epigenetic regulatory mechanisms but also promising therapeutic targets for reprogramming EPCs functionality and enhancing cancer immunotherapy responsiveness.

#### ​​3.2.1 Histone Methylation/Demethylation​​

Histone methylation involves the addition of methyl groups to lysine or arginine residues (including H3K4, H3K27, H3K9) by histone methyltransferases (HMTs such as MLL complexes and EZH2). Its functional outcome depends on the modified site and methylation degree: H3K4me3 typically associates with transcriptional activation, while H3K27me3 and H3K9me3 represent classical repressive marks^34^. At promoter regions of erythroid-specific genes such as the β-globin cluster, H3K4me3 enrichment facilitates transcription factor binding including GATA1 and KLF1 and transcription machinery assembly, driving EPCs toward terminal differentiation. Conversely, non-erythroid lineage genes often accumulate H3K27me3 and H3K9me3 to maintain transcriptional silencing^38^, ^46^. Demethylation is mediated by histone demethylases (KDMs). Lysine-specific demethylase 1 (LSD1/KDM1A) removes H3K4me1/2 marks, thereby suppressing myeloid transcription factor PU.1 expression and preventing EPCs lineage deviation toward myeloid pathways^47^, ^48^. This mechanism is essential for preserving erythroid lineage identity in EPCs. Furthermore, SAMD1 interacts with and potentiates LSD1 activity. Genetic ablation of SAMD1 accelerates erythroid and megakaryocytic differentiation while altering the genome-wide H3K4me2 landscape. In EPCs, SAMD1 co-occupies chromatin with both LSD1 and GATA transcription factors. SAMD1 downregulation reduces global H3K4me2 levels, resulting in transcriptional repression of target genes. Conversely, SAMD1 upregulates transcription at specific genomic loci, demonstrating its dual regulatory function in orchestrating hematopoietic differentiation and EPCs production^49^.

Within the TME, inflammatory factors, hypoxia, and metabolic alterations disrupt HMT-KDM equilibrium. For instance, aberrant EZH2-mediated H3K27me3 deposition may silence critical erythroid genes, while LSD1 dysfunction derepresses myeloid genes including PU.1, promoting acquisition of MDSC-like phenotypes in EPCs^26^. This lineage identity blurring not only suppresses normal hematopoiesis but also enhances tumor immune evasion. Epigenetic agents such as LSD1 inhibitors (tranylcypromine derivatives) and EZH2 inhibitors (tazemetostat) show preclinical potential to restore EPCs differentiation capacity by remodeling histone methylation landscapes, offering intervention strategies for cancer-associated anemia and immunosuppression^50^.

#### ​​3.2.2 Histone Acetylation/Deacetylation​​

Histone acetylation occurs through acetyl group addition to lysine residues (including H3K9 and H3K27) on histone tails by histone acetyltransferases (HATs such as p300/CBP and PCAF)^51^, ^52^. This modification neutralizes lysine positive charges, weakening electrostatic interactions between histones and negatively charged DNA, consequently relaxing nucleosome structure and increasing chromatin accessibility. The resulting physical openness facilitates transcription factor and RNA polymerase II binding at promoters and enhancers, initiating gene transcription. Additionally, acetyl marks recruit transcriptional coactivators and chromatin remodelers via bromodomain-containing proteins (including BRD4), strengthening enhancer-promoter connectivity to amplify transcriptional efficiency^53^, ^54^. During EPCs differentiation, promoters and enhancers of erythroid-specific genes (such as *GATA1* and *KLF1*) accumulate H3K9ac and H3K27ac modifications critical for maintaining high-level erythroid gene expression^55^. Conversely, histone deacetylases (HDACs encompassing classes I, II, and sirtuins) remove acetyl groups, restoring lysine positive charges and promoting chromatin recondensation to suppress transcription. In sickle cell disease models, HDACi induce expression of fetal hemoglobin (HbF) through accumulative activity in EPCs. This process preferentially activates γ-globin gene transcription, elevates acetylated histone H3 levels, and confers an open chromatin conformation at the γ-globin promoter, thereby improving clinical severity and prolonging survival in sickle cell disease patients^56^.

In TME, inflammatory cytokines and oncogenic signals induce aberrant HDAC hyperactivity, reducing acetylation at erythroid genes to block EPCs terminal differentiation and maintain immature immunosuppressive states. In tumor-bearing mouse models, HDAC inhibitors (including entinostat) restore erythroid gene acetylation marks while attenuating immunosuppressive functions of EPCs and MDSCs^45^, indicating potential for reprogramming EPCs differentiation to improve antitumor immunity.

#### ​​3.2.3 Histone Lactylation​​

Tumor cells exhibit a marked metabolic reprogramming characterized by the Warburg effect, whereby they predominantly utilize aerobic glycolysis for energy production, converting glucose to lactate even in the presence of adequate oxygen^57^. This metabolic reprogramming results in markedly elevated lactate concentrations within the TME. Lactate functions not merely as a metabolic waste product but also as a substrate for covalent modification of histone lysine residues, forming lactylation marks (Kla)^58^. This post-translational modification alters chromatin architecture and directly regulates transcriptional programs by modulating transcription factor binding, thereby coupling metabolic states with cell fate decisions. In EPCs regulation, Kla plays a pivotal role: high lactate concentrations in the TME significantly suppress erythroid differentiation, trapping EPCs in immature states while promoting expression of myeloid-suppressive genes and establishing immunosuppressive phenotypes. Mechanistically, lactate modulates lactylation levels at specific histone sites such as H3K14la, activating cell cycle progression and stemness maintenance genes (including *CCNB1* and *CDK6*), while simultaneously repressing erythroid-specific genes such as HBB and ALAS2^59^. Recent therapeutic strategies focused on modulating lactate dynamics, including glycolysis inhibition through LDHA antagonists and lactate clearance via bicarbonate buffering, effectively reduce intracellular lysine lactylation levels. These approaches not only restore physiological endothelial progenitor cell differentiation but also attenuate their immunosuppressive functions, thereby offering innovative avenues for reprogramming EPC-based therapies^60^.

#### ​​3.2.4 Histone Phosphorylation and Ubiquitination​​

GATA1 protein stability and activity are precisely regulated through phosphorylation and ubiquitination. Phosphorylation enhances GATA1 transcriptional activity while simultaneously serving as an ubiquitination signal targeting it for proteasomal degradation, establishing a negative feedback loop for activity termination^61^. SATB1 expression is essential for the upregulation of key erythroid factors HSP70 and GATA1 during MEP differentiation. SATB1 binds to specific sites surrounding the HSP70 locus and facilitates chromatin looping required for HSP70 induction, which in turn promotes GATA1 activation. Although SATB1 expression is progressively downregulated during myelopoiesis, it retains critical biological functions in early-stage EPCs^62^. Under MAPK signaling, coordinated phosphorylation and ubiquitination fine-tune GATA1 protein levels. Molecular chaperone HSP27 further accelerates GATA1 inactivation by promoting its ubiquitination and proteasomal degradation. These rapid, dynamic post-translational modifications ensure quantitative and temporal precision of GATA1 during distinct erythroid differentiation stages, constituting essential mechanisms for maintaining EPCs functional stability^63^.

### 3.3 Regulatory Roles of ncRNAs

miRNAs and lncRNAs modulate EPC functions through post-transcriptional regulation. Specific miRNAs including miR-451 and miR-16 promote erythrocyte maturation, while miR-223 suppresses erythroid differentiation and regulates immunosuppression via STAT3 signaling^64^-^67^. Aberrant miR-223 expression in tumor contexts induces EPCs differentiation blockade. Among lncRNAs, lncRNA-EPS sustains erythroid terminal differentiation by inhibiting the pro-apoptotic gene *Pycard*, whereas *CRNDE204* regulates erythroid/megakaryocytic differentiation through interaction with PUS1 protein^68^. *HOTAIRM1* participates in MDSC immunosuppressive functions. Although direct evidence linking lncRNAs to EPCs phenotypes remains limited, their considerable regulatory potential warrants further investigation^69^.

## 4. EPCs Reprogram Cancer Cell Epigenetic Signatures​​

Conventional perspectives viewed EPCs as passive recipients of TME signals that become trained into immunosuppressive phenotypes. However, emerging research reveals EPCs not only as TME products but also active shapers of the microenvironment. Through secreting soluble factors or releasing extracellular vesicles (EVs), EPCs actively modulate epigenetic states of neighboring cells, establishing a self-reinforcing metabolic-epigenetic-signaling feedback loop^70^. The mechanism involves endothelial progenitor cell-derived EVs serving as intercellular vehicles. These EVs transfer bioactive molecules, including proteins, miRNAs, and lncRNAs, to tumor and immune cells. This transfer facilitates the comprehensive reprogramming of recipient cells, altering their transcriptional profiles and epigenetic states. In tumor contexts, EPCs-secreted EVs are enriched with miRNAs (such as miR-210 and miR-21) and lncRNAs that regulate epithelial-mesenchymal transition (EMT) and cancer stemness^12^, ^71^. Upon uptake by tumor cells, these ncRNAs enhance invasiveness and immune evasion. Furthermore, EPCs-EVs may contain lactylated proteins or RNA molecules that disseminate high-lactate metabolic states to recipient cells, reprogramming their epigenetic configurations to amplify immunosuppressive and pro-tumorigenic microenvironment features. This bidirectional crosstalk not only deepens EPCs-tumor cell synergy but also establishes EPCs as stable, irreversible immunosuppressive modules within the TME^72^. Thus, EPCs transcend their role as mere victims to become active co-conspirators in TME remodeling, playing central roles in immune evasion and tumor progression.

## ​​5. Targeting EPC Epigenetics: Novel Strategy to Enhance Immunotherapy​​

**Figure 3:**
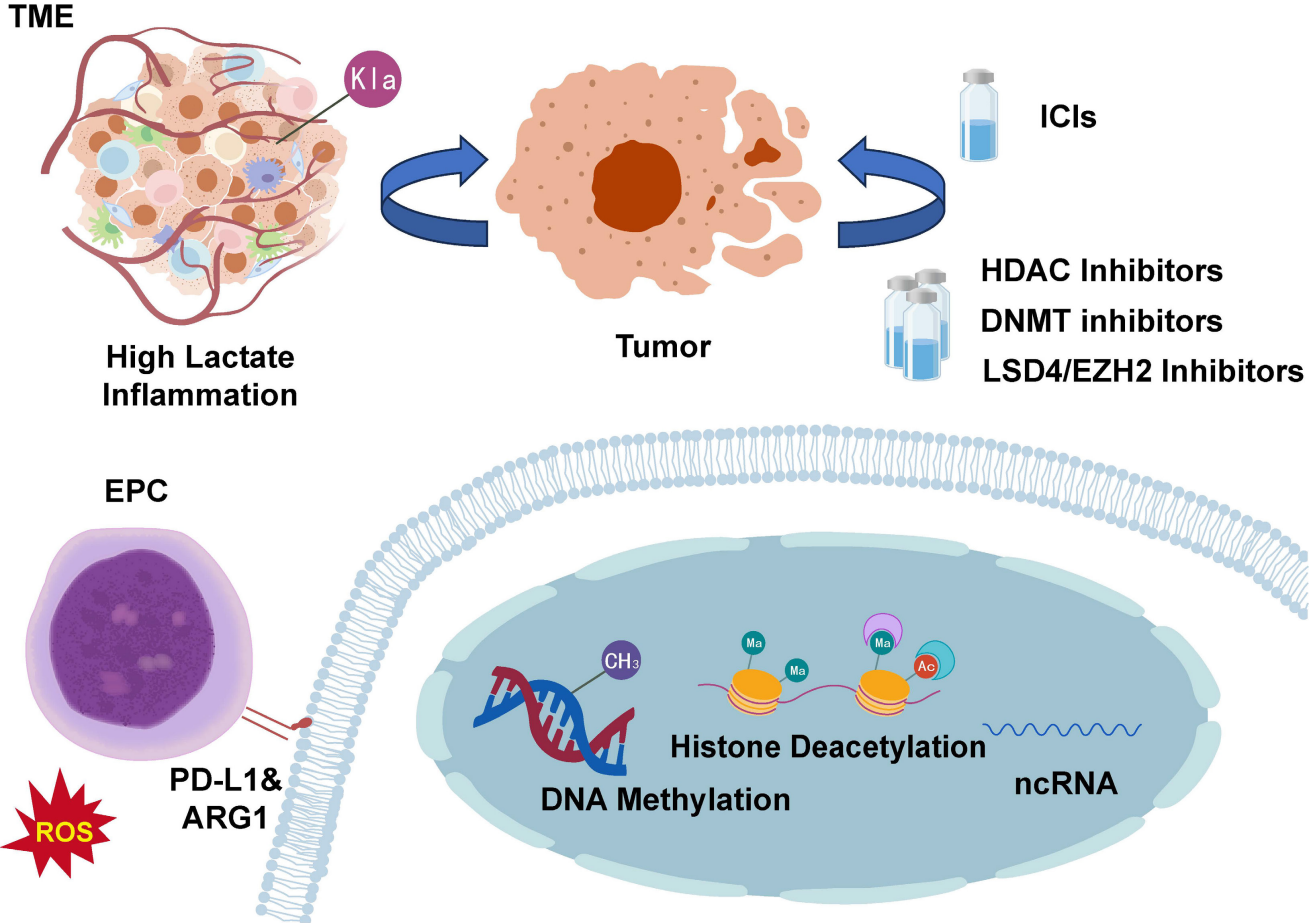
** Targeting Epigenetic Alterations in EPCs​​.** Epigenetic modifications in EPCs can influence cancer progression and represent a significant mechanism underlying cancer therapy resistance. Targeting epigenetic reprogramming in EPCs may improve cancer treatment outcomes and potentially enhance patient survival. EPC, erythroid progenitor cell; TME, tumor microenvironment; ICIs, immune checkpoint inhibitors; ARG1, arginase-1; PD-L1, programmed death-ligand 1; HDAC, histone deacetylase; DNMT, DNA methyltransferase; ncRNA, non-coding RNA; LSD, lysine-specific demethylase.

Despite immunotherapy's transformative impact on cancer treatment, its limited response rates significantly hinder clinical utility. Preclinical and clinical studies demonstrate that combining chemotherapy or targeted therapy with ICIs can overcome resistance in cold tumors^73^, ^74^. EPCs function as critical immunosuppressive components in the tumor microenvironment and demonstrate functional similarity to myeloid-derived suppressor cells, as supported by substantial evidence. These cells display enhanced immunosuppressive capacity through epigenetic mechanisms, suggesting that targeting the epigenetic regulation of EPCs may offer a promising strategy to improve immunotherapy efficacy (**Figure 3**).

### ​​5.1 EPCs Epigenetic Alterations and Immunotherapy Resistance​​

The efficacy of immune checkpoint inhibitor (ICI) fundamentally depends on the presence of reactivatable effector T cells within the TME. However, EPCs that undergo specific epigenetic reprogramming emerge as key contributors to primary and acquired ICI resistance^75^. These EPCs persistently suppress T-cell function through dual mechanisms: overexpression of ARG1 and excessive production of ROS^26^, ^76^. Under combined assault from arginine depletion and oxidative stress, T cells become metabolically impaired and functionally compromised, rendering them incapable of effective proliferation or tumor cell killing despite pharmacological blockade of the PD-1/PD-L1 axis. Moreover, EPCs themselves constitute a major cellular source of PD-L1 within the TME^77^, ^78^. Functioning as endogenous inhibitors, they continuously engage PD-1 receptors on T-cell surfaces, delivering sustained inhibitory signals. During ICI therapy, EPC-derived PD-L1 may competitively bind therapeutic antibodies, reducing blockade efficacy while simultaneously transmitting persistent inhibitory signals to T cells, thereby attenuating overall ICI response intensity^79^-^81^. Mechanistically, aberrant DNA methylation and histone modification patterns cooperatively lock high expression of immunosuppressive genes (including *ARG1* and *CD274*) while desensitizing EPCs to erythroid-differentiation signals such as EPO and GATA1. These epigenetically dysregulated EPCs establish a self-sustaining immunosuppressive barrier within the TME, representing a formidable obstacle to enhancing ICI efficacy^25^. Clinical observations further validate this mechanism, demonstrating significant correlations between elevated EPC abundance in the TME and poor cancer patient prognosis alongside immunotherapy resistance, underscoring their pivotal role in tumor immune evasion.

### 5.2 Epigenetic Drug-Mediated EPCs Reprogramming​

Recent scientific advances have compellingly demonstrated that targeted epigenetic modulators possess the capacity to effectively reverse EPCs dysfunction through multifaceted molecular interventions. **Table 2** summarizes major epigenetic-targeting drugs and their therapeutic potential for targeting tumor-derived EPCs. Specifically, DNA methyltransferase inhibitors such as azacitidine and decitabine operate by demethylating promoter regions of erythroid master regulators including GATA1, while simultaneously modulating the expression profiles of immunosuppressive genes including *ARG1* and *CD274*. DNMTi suppress global DNA methylation in EPCs, leading to derepression of γ-globin genes *HBG1* and *HBG2* with consequent increased fetal HbF expression. In transgenic mouse models of sickle cell disease, oral administration of the DNMTi GSK3482364 significantly elevated HbF levels and the percentage of HbF-expressing erythrocytes while demonstrating favorable overall tolerability^82^, establishing this approach as a promising therapeutic strategy. Furthermore, HDAC inhibitors exemplified by vorinostat, chidamide, and entinostat function through enhancing global histone acetylation levels, thereby activating critical erythroid differentiation programs and suppressing immunosuppressive pathways^83^. Meanwhile, EZH2 inhibitors like tazemetostat achieve therapeutic effects by reducing repressive H3K27me3 histone methylation marks, consequently reactivating silenced erythroid-specific genes essential for proper differentiation^84^, ^85^. Additionally, LSD1 inhibitors including iadademstat correct pathological erythroid-myeloid lineage biases by reducing the accumulation of myelosuppressive EPCs populations through epigenetic reprogramming^86^, ^87^. Collectively, these pharmacologically distinct yet mechanistically complementary agents provide clinically actionable strategies for precision-targeted EPCs therapy while simultaneously creating novel synergistic opportunities when integrated with contemporary immunotherapeutic regimens.

### 5.3 Combination Therapies with Multi-Target Epigenetic Drugs

Given the multifactorial nature of epigenetic reprogramming, combination therapies or single agents with multi-target specificity offer promising strategies to redirect dysregulated disease-associated molecular networks for improved therapeutic efficacy^88^. Multi-target epigenetic intervention can be readily achieved by administering two or more individual inhibitors, either to enhance the effect of a single agent or to overcome resistance to a specific epigenetic drug^89^. While HDACi used as monotherapies have shown limited benefit in early-phase clinical trials for myeloproliferative neoplasms, subsequent studies in multiple cancer cell lines revealed that sequential administration of HDACi following DNMT inhibition synergistically enhanced the expression of silenced tumor suppressors and promoted cell death^90^, ^91^. This concept has been validated in several clinical trials (NCT00275080, NCT01130506, and NCT00882206), which primarily evaluated decitabine in combination with vorinostat.

In multi-drug regimens, drug combinations may influence the same or distinct cellular pathways or epigenetic mechanisms. Inhibitors can act independently, or the activity of one drug may potentiate the efficacy of another^92^. However, divergent pharmacokinetic and dynamic properties among combined agents may hinder synergistic or additive effects. Despite the clear potential of epigenetic drug combinations, several challenges remain. Although proof-of-concept clinical trials are advancing rapidly, all potential drug interactions require systematic evaluation. While the efficacy of single agents is a prerequisite for their use, head-to-head comparisons between combination and monotherapy regimens are often necessary to rule out combination-specific toxicities or side effects not observed with individual drugs^93^. Since all molecules in a combination have known molecular targets, identifying unexpected inter-drug interactions within disease-relevant pathways and detecting synergistic or additive effects become essential. Furthermore, the co-administration of chemically disparate molecules complicates pharmacokinetic and pharmacodynamic optimization. A promising direction may lie in designing personalized treatment strategies that balance patient-specific conditions and disease-associated molecular profiles. Single-target and multi-target approaches should not be viewed as competing, but rather as complementary therapeutic avenues^94^.

### ​​

## ​​6. Conclusions and Perspectives​​

EPCs serve as essential precursors in erythropoiesis, maintaining erythroid differentiation homeostasis under physiological conditions through precise epigenetic regulation, including DNA methylation, histone modifications, and non-coding RNA-mediated control. Accumulating evidence reveals their significant contributions to tumor immunosuppression and the profound impact of aberrant epigenetic reprogramming on antitumor immunity. Although current research on epigenetic regulation of EPCs remains in its developmental stages with mechanistic understanding still evolving, existing studies demonstrate that EPCs differentiation and functional maintenance involve multiple epigenetic mechanisms, including DNA methylation, histone modifications, and ncRNAs regulation^11^. These mechanisms not only function during hematopoietic homeostasis but also undergo reprogramming in the TME. Tumor-derived factors can alter DNA methylation patterns or histone modification profiles in EPCs, thereby influencing their immunomodulatory functions and differentiation trajectories. Moreover, non-coding RNAs exhibit potential regulatory roles in the interactions between EPCs, immune cells, and tumor cells. Targeting these pathological epigenetic states in EPCs offers a promising therapeutic strategy. Epigenetic drugs can reprogram EPCs to restore normal erythroid differentiation potential while attenuating immunosuppressive phenotypes, potentially revitalizing immunological activity within the TME. However, systematic studies on EPC epigenetic signatures remain relatively scarce, particularly regarding differential regulatory mechanisms across various tumor types and disease states. Future research should integrate single-cell omics, multi-omics analyses, and functional validation to comprehensively decipher the EPCs epigenetic regulatory network and its potential value in cancer immunotherapy.

### ​​​​6.1​​ Deciphering the mechanistic basis of epigenetic reprogramming in EPCs

Research on epigenetic modifications and regulatory mechanisms in EPCs remains relatively limited and requires further comprehensive investigation. A primary challenge lies in the incomplete mechanistic understanding of EPCs epigenetic regulation, necessitating integrated multi-omics approaches combined with artificial intelligence (AI) innovations to elucidate the molecular networks governing EPCs epigenetics. Encouragingly, the research focus in this field is gradually shifting from describing single epigenetic markers to analyzing multi-layered, multidimensional epigenetic networks and their dynamic regulation. On one hand, integrating multi-omics data to construct comprehensive epigenetic regulatory maps aims to reveal interaction patterns among different modification types and their contributions to tumor heterogeneity^95^-^97^. On the other hand, developing more targeted and reversible epigenetic editing tools has become a research hotspot, with the goal of achieving precise intervention in tumor-specific phenotypes while minimizing toxicity. Furthermore, understanding how epigenetic mechanisms regulate tumor immune evasion pathways within the immune microenvironment has become essential for developing more effective immunotherapeutic strategies. AI algorithms could precisely identify potential regulatory nodes within each omics layer that contribute to epigenetic reprogramming, enabling accurate prediction of factors driving aberrant epigenetic states in EPCs and establishing foundations for epigenetics-based targeted therapies. AI-assisted epigenetic drug design has achieved considerable success, with several candidates advancing to preclinical development^98^, ^99^. However, research on pharmacological interventions targeting epigenetic regulation in EPCs remains limited. Notably, AI-driven approaches may accelerate the development of therapeutics targeting EPC epigenetic reprogramming^100^. Although preliminary 3D chromatin landscapes during erythroid progenitor differentiation have been constructed^101^, substantial exploration is still required regarding multi-omics application scenarios, combinatorial strategies, and model refinement.

Additionally, accelerating the development and optimization of preclinical models remains imperative to validate EPCs functions and epigenetic alterations within TME using clinically relevant systems. Current animal models exhibit significant limitations in recapitulating pathological EPCs recruitment, differentiation, and immunomodulatory effects in human tumors. Murine tumor cell-based models particularly demonstrate substantial epigenetic divergences between murine and human EPCs biology. While patient-derived xenograft (PDX) models mitigate tumor cell-intrinsic discrepancies, immune rejection or recognition of human tumor cells as foreign entities introduces instability in epigenetic influences on EPCs. Future model development must focus on constructing systems that more accurately replicate the dynamic interactions among EPCs, immune cells, and tumor cells while faithfully capturing epigenetic interactions between EPCs and malignancies. Developing personalized models incorporating patient-specific tumor microenvironment variations will be essential for reliably assessing the therapeutic efficacy of EPCs-targeted epigenetic reprogramming strategies across individual patients.

### ​​6.2​​ Identification of biomarkers associated with EPC epigenetic dysregulation

Accurately evaluating the extent of epigenetic reprogramming in EPCs constitutes a critical prerequisite for refining epigenetic-targeted therapeutic approaches. Current EPCs definitions predominantly rely on surface markers, with CD71 and CD235a serving as primary identifiers in humans, while CD71 and Ter119 are utilized in murine systems. However, significant challenges arise due to phenotypic heterogeneity of EPCs across different pathological states and overlapping marker expression with other cell populations, complicating precise EPC identification. To address this limitation, integrating single-cell sequencing technologies to decipher EPCs transcriptomic signatures could enable the screening of subtype-specific molecular markers. Concurrent application of mass cytometry and spatial imaging techniques would further permit *in situ* identification and functional classification of distinct EPCs subpopulations within tissues. Such multidimensional characterization would establish biomarkers reflecting epigenetic modifications or reprogramming events in TME-resident EPCs, thereby enabling not only assessment of reprogramming extent but also monitoring of continuous progression and dynamic evolution of epigenetic alterations. Liquid biopsy represents a promising non-invasive strategy for biomarker detection^102^; however, given the predominant hepatic localization of EPCs, substantial validation is required to determine whether conventional blood or bodily fluid samples can adequately capture epigenetic reprogramming status in these cells. From a translational perspective, epigenetic research has facilitated the development of novel diagnostic and therapeutic strategies. Liquid biopsy techniques based on circulating free DNA (cfDNA) methylation profiles have demonstrated high sensitivity and specificity for early cancer detection and recurrence monitoring in certain malignancies. Epigenetic drugs such as DNA methyltransferase inhibitors (DNMTis) and histone deacetylase inhibitors (HDACis) have received clinical approval for specific indications and show promising synergistic potential when combined with immunotherapy or targeted therapy^103^, ^104^. Future investigations should further elucidate the epigenetic regulatory mechanisms governing EPCs, specifically defining aberrant epigenetic control of individual genes across diverse pathological contexts and clarifying their potential impacts on tumor immunity and precise roles in immunoregulatory networks. Such insights would provide the foundational rationale for developing targeted interventions.

### ​​6.3​​ Targeting EPC epigenetics to enhance cancer immunotherapy

EPCs exert substantial influence on the tumor microenvironment through their epigenetic modifications, which may promote immunosuppressive TME formation or potentially reverse immunologically cold tumor phenotypes. Targeting these epigenetic alterations in EPCs thus represents a promising strategy to enhance the efficacy of cancer immunotherapy. Epigenetic drugs and immunotherapies exhibit synergistic antitumor effects, as demonstrated by clinical evidence in classical hodgkin lymphoma (cHL) where the combination of decitabine and the immune checkpoint inhibitor camrelizumab achieved significantly higher complete response (CR) rates compared to camrelizumab monotherapy. This combination may overcome resistance to PD-1 inhibitors in cHL patients^105^.

Collectively, diverse classes of epigenetic agents and multiple clinical cases provide robust support for cancer epi-immunotherapy. However, EPCs-targeted epigenetic drugs face substantial challenges. Specificity issues and off-target effects severely hinder clinical translation; for example, while HDAC inhibitors can suppress MDSC-mediated immunosuppression, most are pan-inhibitors affecting multiple HDAC isoforms, thereby inducing toxicity in normal cells. Additional limitations include poor sensitivity in solid tumors, prolonged timeframes required for epigenetic landscape reprogramming, and low selectivity for histone versus non-histone targets, restricting their application in solid tumor clinical management. Nanomedicine strategies offer promising approaches to attenuate toxicity while enhancing efficacy. Delivery systems including nanoparticles, liposomes, dendrimers, and nanogels, when engineered with prodrugs or combination therapies, can significantly minimize off-target toxicity^106^. Nevertheless, both epigenetic drugs and their delivery platforms remain predominantly in preclinical development stages, necessitating further investigation.

**Figure 4:**
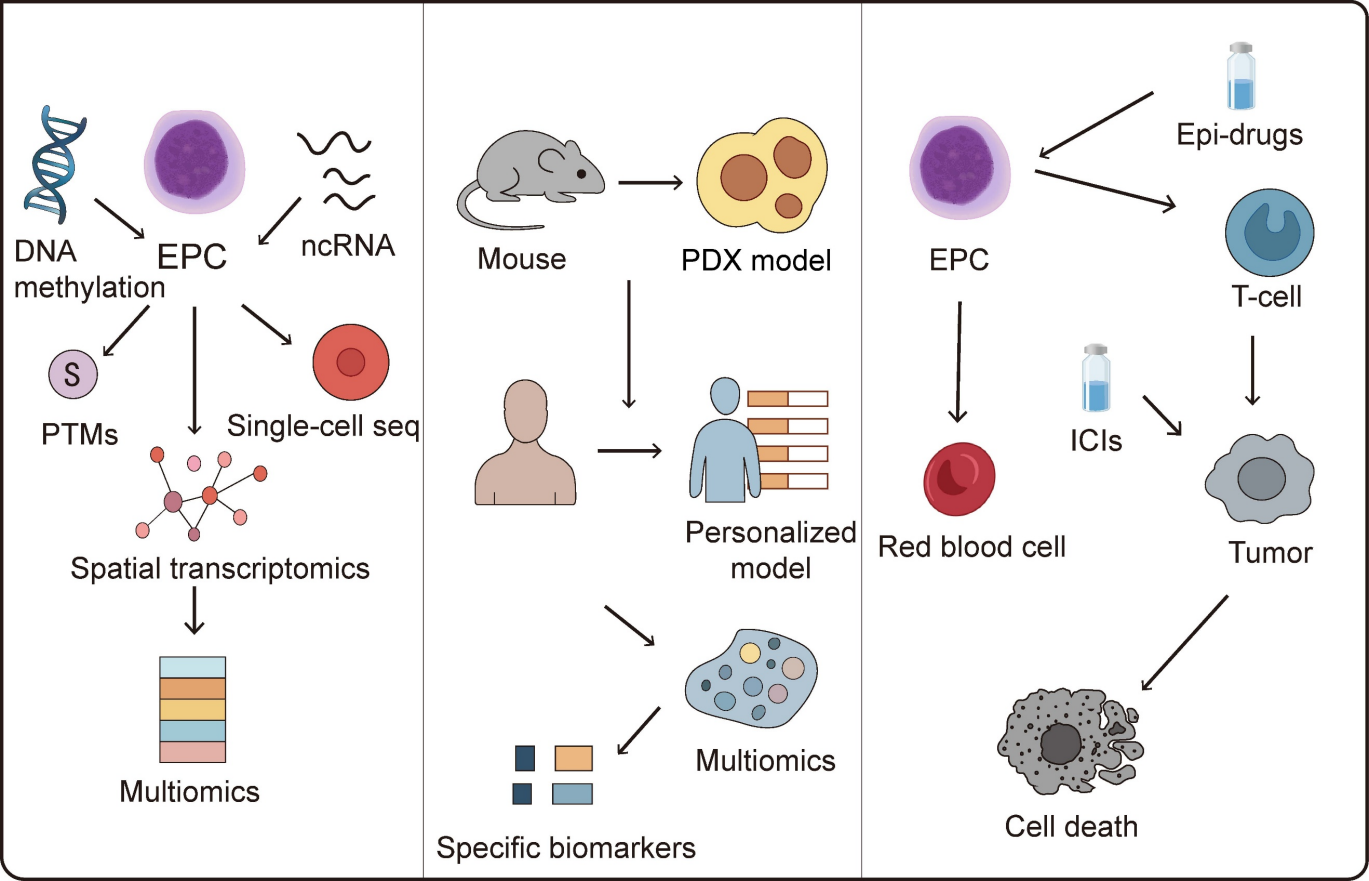
** Epigenetic Modifications of EPCs and Cancer Immunotherapy​**​. A deeper understanding of the mechanisms underlying epigenetic alterations in EPCs may lead to the identification of novel biomarkers and offer opportunities to enhance immunotherapy efficacy through targeted epigenetic reprogramming of EPCs. EPC, erythroid progenitor cell; ICIs, immune checkpoint inhibitors; PTMs, post-translational modifications; PDX, patient derived xenograft.

Ultimately, resolving fundamental questions about EPCs epigenetics through mechanistic dissection, advanced modeling, and biomarker discovery will illuminate novel pathways to enhance cancer immunotherapy (**Figure 4**). Future research must establish comprehensive epigenetic atlases of EPCs across tumor types, develop clinically relevant biomarkers for reprogramming assessment, and optimize combination strategies between epigenetic modulators and immunotherapies. These advances hold transformative potential for overcoming immunotherapy resistance and improving survival outcomes for cancer patients.

## Figures and Tables

**Table 1:** Major Epigenetic Modifications and Their Functions in EPCs

Modification Type​​	​​Key Enzymes (Writers/Erasers)​​	​​Role in Normal Erythropoiesis​​	​​Aberrant Modification in Tumor-Associated EPCs​​	​​References​
DNA Methylation​​	DNMT1 / TET2	Silences non-erythroid genes; maintains lineage specificity	Imbalanced hyper/hypomethylation; disrupted by TME lactate metabolism; interferes with erythroid differentiation	35, 37
​​H3K27 Acetylation​​	p300 / HDACs	Activates erythroid enhancers; promotes erythroid gene expression	Imbalanced acetylation/deacetylation; silences key erythroid genes	52, 55
​​H3K4 Demethylation​​	LSD1 (KDM1A) / JMJD3	Removes activation marks from myeloid transcription factors; maintains erythroid fate	Increases EPCs lineage plasticity; promotes myeloid characteristics	26, 49
​​H3K27 Trimethylation​​	EZH2 / KDM6A (UTX)	Suppresses non-erythroid genes via H3K27me3; HSC self-renewal and erythroid differentiation	EZH2 hyperactivation induces erythroid suppression; interacts aberrantly with TME signals	46, 50
​​H3K14 Lactylation​​	Lactyltransferase (putative) / Unknown	Delays erythroid differentiation; maintains progenitor activity	Elevated lactate in TME upregulates H3K14la; accumulates immature EPCs	59
​​GATA1 Ubiquitination​​	E3 ligase / Deubiquitinase	Regulates GATA1 stability; limits activity cycles; ensures stage-specific differentiation	Dysregulated ubiquitination/degradation dynamics; disrupts erythroid gene expression and EPCs function	37, 62
​​Phosphorylation​​	Aurora B / JAK2	Regulates mitosis and signal transduction	Hyperactivated JAK2/STAT5 pathway promotes erythroid expansion; abnormal phosphorylation disrupts differentiation	107
​​SUMOylation​​	Ubc9 / PIAS1	Stabilizes transcription complexes; fine-tunes erythroid differentiation	Excessive SUMOylation inhibits pro-erythroid gene transcription	108
​​Non-coding RNA Regulation​​	lncRNA-EPS	Maintains erythroid developmental homeostasis	TME-induced miRNA imbalance suppresses erythroid gene expression	68

**Table 2:** Epigenetic Drugs for Reprogramming Tumor-Associated EPCs

Drug (Representative)	Drug Class	Primary Molecular Target/Mechanism	Potential Impact on EPCs/Tumor-Associated EPCs	Clinical/R&D Stage and Key Considerations	​​References​
Azacitidine / Decitabine	DNMT inhibitors (DNMTi)	Inhibit DNMTs; induce demethylation and gene reactivation	Restore erythroid TFs; promote differentiation; reduce immature EPCs; downregulate ARG1/PD-L1; weaken EPC immunosuppression	Approved for MDS/AML; potential EPC reprogramming	82
Vorinostat, Chidamide, Entinostat	HDAC inhibitors (HDACi)	Block histone deacetylation; increase H3K9ac/H3K27ac; open chromatin	Promote erythroid programs; relieve differentiation arrest; attenuate EPC/MDSC-mediated suppression; enhance ICI efficacy	Approved/clinical trials with ICIs; caution: pan-HDACi toxicity, dual immune effects; dosing/timing critical	54, 56
Tazemetostat	EZH2 inhibitor	Inhibit PRC2/EZH2; reduce H3K27me3-mediated silencing	Restore erythroid gene expression; reduce immunosuppressive silencing; effects vary by differentiation stage	Approved for lymphomas; EPC use requires biomarker-guided monitoring	26, 83
Seclidemstat	LSD1 inhibitor (KDM1A)	Block LSD1-mediated demethylation (H3K4me1/2, H3K9me1/2)	May shift EPCs toward myeloid fate; impair differentiation; risk of enhanced suppressive phenotypes	In trials for solid tumors/fusion cancers; EPCs application risky; requires validation and combination strategies	87
Combination Strategies	Multi-target epigenetic modulators	Reshape methylation/acetylation networks; enhance transcriptional reprogramming	Synergistically restore erythroid transcription; reduce immunosuppressive genes	Preclinical/early trials with ICIs show synergy; caution: toxicity	92, 94

## Abbreviations

EPC: erythroid progenitor cell
TME: tumor microenvironment
ROS: reactive oxygen species
MDSC: myeloid-derived suppressor cell
Treg: regulatory T cell
ARG-1: arginase-1
TGF-β: transforming growth factor-beta
TAM: tumor-associated macrophage
ncRNA: non-coding RNA
miRNA: microRNA
lncRNA: long non-coding RNA
HSC: hematopoietic stem cell
MPP: multipotent progenitor
CLP: common lymphoid progenitor
CMP: common myeloid progenitor
GMP: granulocyte-macrophage progenitor
MEP: megakaryocyte-erythroid progenitor
BFU-E: burst-forming unit-erythroid
CFU-E: colony-forming unit-erythroid
EMH: extramedullary hematopoiesis
PDGF-B: platelet-derived growth factor-B
EDMC: erythroid-derived myeloid cell
PD-L1: programmed death-ligand 1
PD-1: programmed death protein 1
HDAC: histone deacetylase
DNMT: DNA methyltransferase
HDACi: histone deacetylase inhibitor
DNMTi: DNA methyltransferase inhibitors
AID: activation-induced cytidine deaminase
LSD: lysine-specific demethylase
m6A: N6-methyladenosine
EV: extracellular vesicle
EMT: epithelial-mesenchymal transition
ICI: immune checkpoint inhibitor
cfDNA: circulating free DNA
PDX: patient-derived xenograft
AI: artificial intelligence
CR: complete response
cHL: classical hodgkin lymphoma
